# Supplementary figures and images for: Using Sentinel-1, Sentinel-2, and Planet satellite data to map field-level tillage practices in smallholder systems
Source: PLoS One. 2022 Nov 28;17(11):e0277425. doi: 10.1371/journal.pone.0277425 (PMC9704639; doi:10.1371/journal.pone.0277425)

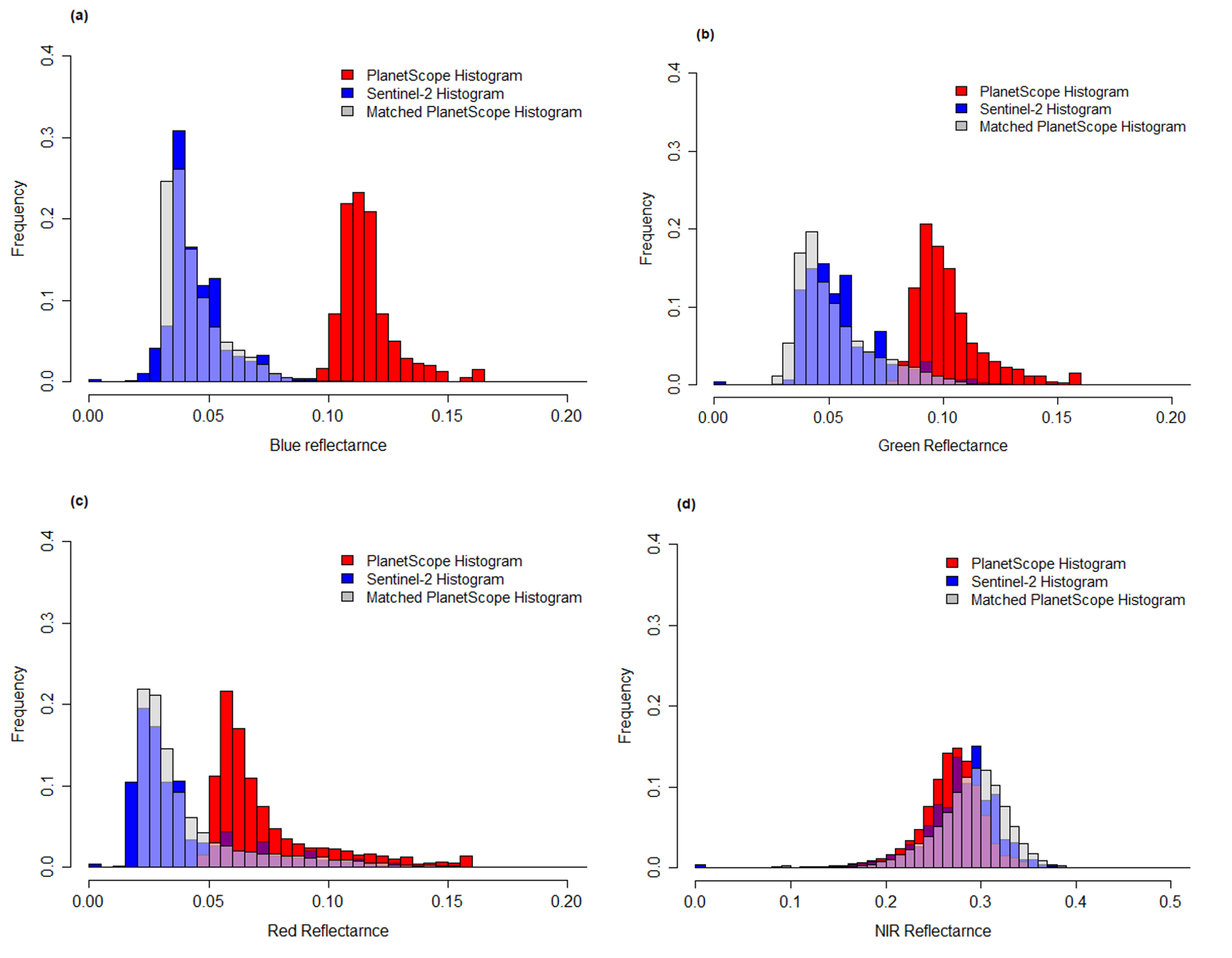

Supplement: S1 Fig — Histograms for Sentinel-2 (shown as blue), raw PlanetScope (shown as red), and histogram-matched PlanetScope (shown as grey) for images from October 8, 2017 for the (a) Blue, (b) Green, (c) Red, and (d) NIR bands. (TIF) [file pone.0277425.s001.tif]

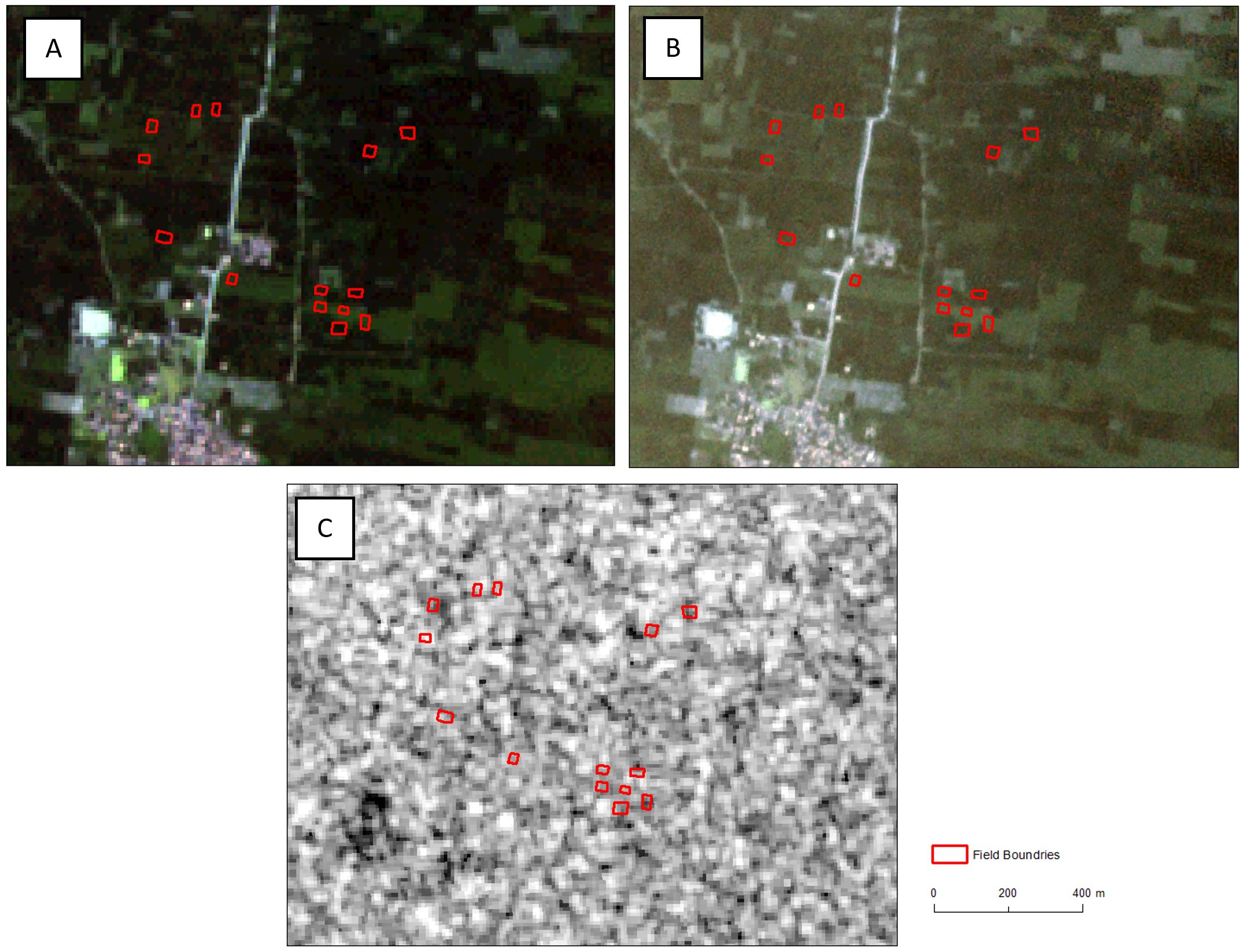

Supplement: S2 Fig — (TIF) [file pone.0277425.s002.tif]
