# Supplementary material for: Using Sentinel-1, Sentinel-2, and Planet satellite data to map field-level tillage practices in smallholder systems
Source: PLoS One. 2022 Nov 28;17(11):e0277425. doi: 10.1371/journal.pone.0277425 (PMC9704639; doi:10.1371/journal.pone.0277425)
Supplement: S1 File — (DOCX) [file pone.0277425.s003.docx]

**Supplementary Information**

**Table S1.** **Survey conducted with farmers in the study** (we only collect this information for wheat plots).

**1. General Information (All crop type fields)**

| Farmer Full Name |  |
| --- | --- |
| Father’s Full Name |  |
| Phone Number |  |
| Village, Block |  |

**2. Plot Coordinates (All crop type fields)**

|  | Corner 1 | Corner 2 | Corner 3 | Corner 4 | Center |
| --- | --- | --- | --- | --- | --- |
| N |  |  |  |  |  |
| E |  |  |  |  |  |

**3. Crop Residue & Soil Till Information (Only collect this information for wheat plots)**

| Did you till the soil before planting wheat? | | Yes  No |
| --- | --- | --- |
| If yes: | How many days before seeding wheat did you till your field? |  |
|  | What did you use to till your soil (circle all applicable choices)? | Rotovator  Plough/Harrow  Planking  Other ________________ |
|  | What was the number of passages you used for each method? | Rotovator _________  Plough/Harrow _________  Planking ____________  Other _________________ |
|  | What type of tractor did you use to till your soil? | My own tractor  Rented tractor  Other _____________ |
| Did you irrigate your wheat plot before sowing? | | Yes  No |
| If yes: | How many days before seeding did you irrigate your wheat? |  |
| Which crop was grown before wheat seeding? | |  |
| How was this previous monsoon crop harvested? | | Manual  Combine  Other _________________ |
| How was the previous monsoon crops’ residue managed? | | Collected  Burnt  Other _________________ |
| What crop will you plant in this plot during the upcoming monsoon season? | |  |
| Will you till the soil before planting this monsoon crop? | | Yes, I already tilled  Yes, I will till later  No, I will not till |
| If yes: | Will you do puddling before planting the monsoon crop, and is it deep or light? | Yes, I do deep puddling  Yes, I do light puddling  No, I do not do puddling |
